# Sex- and Age-Specific Characteristics of Running Performance Assessed by OptoJump in Pre-School Children Aged 3 to 6 Years

**DOI:** 10.3390/children12121684

**Published:** 2025-12-11

**Authors:** Sanja Ljubičić, Jera Gregorc, Vilko Petrić

**Affiliations:** 1 Faculty of Teacher Education, University of Rijeka, 51000 Rijeka, Croatia; vilko.petric@ufri.uniri.hr; 2 Faculty of Education, University of Ljubljana, 1000 Ljubljana, Slovenia; jera.gregorc@pef.uni-lj.si

**Keywords:** children, motor patterns, propulsive phase, running performance, spatiotemporal characteristics, temporal–kinematic

## Abstract

**Highlights:**

**What are the main findings?**

- Our research quantifies fundamental running characteristics in children aged 3 to 6 years using the OptoJump system, showing how speed, step length, and propulsive phase change with age.
- The study identifies sex-dependent patterns in running dynamics, emphasizing the variability and developmental progression of motor skills in early childhood.

**What are the implications of the main findings?**

- The findings provide reference data that can guide future research on motor development in early childhood.
- Policymakers and practitioners can use these data to promote healthy motor development and prevent physical inactivity in preschool children.

**Abstract:**

**Background/Objectives:** Running is among the most prevalent forms of physical activity in preschool-aged children and constitutes a fundamental component for the effective execution of other motor patterns. The main aim of this study is to determine how fundamental running parameters change with age and whether there are differences between sexes. **Methods:** Four-hundred and five pre-school children with the mean (SD) age = 4.9 (1.1) years, height = 111.2 (9.3) cm, weight = 20.0 (4.2) kg, 53.5% girls were recruited from 34 kindergartens in four major cities. The inclusion criteria involved children aged 3–6 years with typical development and without any locomotor or mental disorders and diseases, who were enrolled in day care. Running performance was assessed in preschool children using a 10-m sprint test. Sprint parameters were measured with the OptoJump modular system, an infrared platform that accurately quantifies kinematic variables. Sex (boys vs. girls) and age (3 to 6 years old) differences were calculated by using analysis of variance (ANOVA) or Kruskal–Wallis H-test with post hoc comparison test between the groups. **Results:** In general, the results indicated that statistically significant differences between boys and girls were observed across the following levels: (1) temporal–kinematic step phase, (2) spatiotemporal movement characteristics, and (3) propulsive phase as an indicator of muscular activity. However, these differences were not consistent across all age groups. **Conclusions:** This study provides new insights into the spatiotemporal characteristics of running in preschool-aged children. The findings may assist in the early identification of potential motor deviations and in the planning of more effective strategies to promote physical activity during the preschool period.

## 1. Introduction

The preschool period represents a critical phase for the development of fundamental locomotor skills, including running, which contributes to cardiovascular health and physical fitness [1,2,3]. Running typically emerges during the second year of life, when neuromuscular maturation reaches a sufficient level [4]. Childhood is a sensitive period for shaping motor competence, which is associated with later engagement in sports and physical activity [5,6]. Insufficient levels of motor competence and physical activity during the preschool years are linked to an increased risk of obesity, reduced cardiorespiratory endurance, and poorer cognitive and social outcomes [7,8,9].

Running is one of the most common forms of physical activity among preschool children [10] and represents an essential activity for the successful execution of other motor patterns. It is characterized by a cycle consisting of two steps, each including two support phases (stance phases) and two non-support phases (flight phases) [11]. The assessment of running performance is widely used for talent identification and the evaluation of physical fitness in both sports and educational settings. Examining differences in specific sociodemographic characteristics, such as sex and age, is useful for accurately monitoring running parameters, as it enables the identification of children with lower motor competence and the determination of whether a child’s motor development is typical for their age. Although most children (70–75%) at the age of four are capable of mastering the skill of running [5], previous research indicates significant interindividual differences in running performance [2]. In other words, there is no uniform running pattern, and each child performs the movement significantly differently from the average. Relatively few studies have analyzed age- and sex-specific characteristics of running in early and preschool-aged children in detail [12,13,14]. Existing literature shows that average running speed (over 20 m) increases significantly with age: at 3 years it was 2.89 m/s, at 4 years 3.21 m/s, at 5 years 3.45 m/s, and at 6 years 3.64 m/s [12]. In contrast, one study indicates that vertical loading rate during the push-off phase decreases by approximately 31% with age, suggesting improved shock absorption and push-off strength [14]. Research examining sex differences in the temporal–kinematic phases of running indicates that preschool girls exhibit more stable temporal and biomechanical structures compared to boys [13,15]. Conversely, some authors report that preschool boys achieve better force absorption and superior biomechanical control during the push-off phase [14]. Differences in running dynamics can likely be attributed to the development of the neuromotor system and the efficiency of the lower-limb stretch-shortening cycle (SSC). Preschool children still demonstrate inconsistent patterns of muscle activity, most notably in eccentric muscle contractions during the push-off and landing phases [16]. In this age group, the maturation of brain and perceptual functions outpaces the development of the neuromuscular system; while cognitive and perceptual components of movement develop more rapidly, the integration of the nervous and muscular systems occurs more gradually [17].

In educational and pedagogical practice, traditional measurement methods are most commonly applied, whereas modern systems such as OptoJump allow for precise and rapid analysis of parameters including step length, running speed, ground contact time, flight phase duration, and others. In this context, early monitoring and understanding of developmental characteristics of running in preschool children hold significant public health relevance, as they enable the timely identification of children at risk and the implementation of targeted interventions to promote healthy lifestyle habits. Therefore, the primary aim of this study is to determine how fundamental running parameters change with age and whether there are differences between sexes.

## 2. Materials and Methods

### 2.1. Study Participants

In this observational, cross-sectional study, 405 preschool children (mean age 4.9 ± 1.1 years; height 111.2 ± 9.3 cm; weight 20.0 ± 4.2 kg; 53.5% girls) were recruited from kindergartens in Rijeka and Zagreb (Croatia) and Ljubljana and Koper (Slovenia). Participants were aged 3–6 years, typically developing, without diagnosed locomotor or cognitive impairments, and enrolled in day care programs. A priori sample size estimation using G*Power (version 3.1.9.7) (ANCOVA, 2 groups: boys vs. girls; 1 covariate: age; power 0.80; α = 0.05; n = 405; f = 0.20) indicated that small to medium group differences could be detected. Investigators supervised data collection directly; however, all data were anonymized using unique participant codes. Children were not randomly assigned; groups were defined by naturally occurring characteristics (age and sex). Parents/guardians provided written consent, and the study complied with national regulations and the Helsinki Declaration [18], approved by the Faculty of Teacher Education, University of Rijeka (Ethical approval no. uniri-mladi-drustv-23-37: KLASA:641-01/24-01/03; URBROJ: 2170-1-38-10-24-2; uniri-iskusni-drustv-23-201: KLASA: 641-01/24-01/03; URBROJ: 2170-1-38-10-24-1). Data collection took place from 17 May to 19 November 2024

### 2.2. Running Performance

To assess running performance, we employed the OptoJump modular system (Microgate, Bolzano, Italy), an infrared platform composed of two parallel bars spaced 1 m apart. Each bar is equipped with light-emitting diodes that detect any interruption in communication, and the system is interfaced with a personal computer via specialized software to quantify running parameters. The OptoJump system has demonstrated strong validity and reliability, with very high validity for kinematic parameters (ICC = 0.96–0.99; mean bias = 0.4–2.7%) and high accuracy in the assessment of step and temporal variables [19].

The 10-m sprint test was conducted with preschool-aged children under controlled conditions. The testing was conducted indoors, during the morning hours, specifically between 9:00 a.m. and 12:00 p.m., ensuring consistent conditions for all children. The testing was administered by kinesiologists with expertise in children’s motor development. All children ran in age- and foot-size-appropriate athletic footwear (sneakers) to ensure safety and standardized testing conditions. A 10-m distance was marked with tape on a flat and unobstructed surface [20], indicating the start and finish lines. To minimize the impact of deceleration on running performance, a 2-m OptoJump system was placed at the center of the 10-m distance to obtain relevant biomechanical parameters. Participants performed three maximal 10-m acceleration sprints from a standing start, during which they were allowed to choose which leg to place forward in the starting position [21]. Participants received verbal instructions to stand with their toes on the starting line (high start position) and to run as fast as possible, without compromising safety, to the finish line [20]. One of the evaluators walked alongside the track to monitor the participants’ movement for safety purposes. For further analysis, the best sprint test (0–10 m) was used, i.e., the one in which ground contact time was the shortest. The Optojump software (version 1.13.24.0) generated data regarding contact time (s, %), flight time (s, %), swing phase (s), stride time (s), elevation (cm), speed (m/s), acceleration (m/s^2^), step (cm), stride (cm), pace (steps/s, steps/min), contact phase (s, %), foot flat phase (s, %), propulsive phase (s, %).

### 2.3. Data Analysis

Data normality was calculated using the Kolmogorov–Smirnov (K-S) test. Basic descriptive statistics are presented as mean (standard deviation; SD) or as median and interquartile range (25th and 75th percentile) for normally and not-normally distributed variables. Sex (boys vs. girls) and age (3 to 6 years old) differences were calculated by using analysis of variance (ANOVA) or Kruskal–Wallis H-test with post hoc comparison test between the groups. Main effects for ‘sex’, ‘age’ and the ‘sex * age’ interaction term were determined. If significant main effects occurred, differences between the groups were examined using Student’s *t*-test for independent samples. All analyses were performed in Statistical Packages for Social Sciences version 24. (SPSS Inc., Chicago, IL, USA).

## 3. Results

Basic descriptive statistics of the study participants are presented in Table 1.

Main effects for ‘sex’ showed significant sex differences in swing and stride phases, speed, pacing, contact phase, foot flat and the percentage in propulsive phase (*p* < 0.05). Specifically, boys outperformed girls in speed (3.4 vs. 3.2 m/s, *p* = 0.005), pacing (4.3 vs. 4.0 steps/s, *p* < 0.001) and steps/m (255.5 vs. 241.2 steps/min, *p* < 0.001) and propulsive phase (%, 46.5 vs. 43.5%, *p* = 0.009). Girls exhibited higher values in swing phase (0.31 vs. 0.29 s, *p* < 0.001), stride time (0.49 vs. 0.46 s, *p* < 0.001), contact phase (0.019 vs. 0.015 s, *p* < 0.001) and the percentage of contact phase (9.9 vs. 8.3%, *p* < 0.001) and foot flat phase (0.085 vs. 0.079, *p* = 0.004). When assessing main effects for ‘age’, the percentage of contact and flight times, foot flat phase and the percentage of foot flat phase significantly decreased by age, while increases in speed, acceleration, step and stride lengths, propulsive phase and the percentage of propulsive phase with age were observed (*p* < 0.05). Main effects for ‘sex × age’ interactions did not reveal significant differences between the study groups, indicating that sex did not influence on age and vice versa, when observing running performance outcomes (*p* > 0.05).

## 4. Discussion

This study analyzed the spatiotemporal characteristics of running in children aged 3–6 years to examine age-related changes and sex differences in fundamental running parameters. Group variability (homogeneity) was assessed as an indicator of running pattern maturity, with higher variability reflecting less mature and more diverse movement strategies, and lower variability indicating a more coordinated, stable, and efficient motor control [22].

When interpreting the results, it is important to acknowledge that several factors inherent to field-based testing in preschool children cannot be fully standardized. These include differences in surface characteristics and footwear, variations in the intensity of verbal encouragement, and the child’s momentary motivation or emotional state, which can be influenced by interventions and structured activities designed to engage preschoolers [23] (Boobani et al., 2025). Such contextual influences may partly account for the observed inter-individual differences in the kinematic phases of the running cycle and thus provide an important framework for understanding the outcomes. We first addressed developmental changes in the biomechanical and spatiotemporal characteristics of running during the preschool period. Particular focus was placed on changes occurring between ages 3 and 6, which were associated with neuromotor maturation, improvements in neuromuscular coordination, and the gradual transition to more energy-efficient and stable movement patterns. Subsequently, we analyzed sex differences in specific running parameters. These differences are interpreted as potential consequences of variations in body composition, muscular strength development, and the use of different stabilization strategies. The final part of the discussion considers the potential interaction effect between sex and age, focusing on whether the developmental dynamics of selected parameters differ between boys and girls and whether sex-related differences are expressed as stable characteristics independent of chronological age.

Our results indicated that children aged 3 to 6 years showed improvements in spatiotemporal and biomechanical running characteristics. Specifically, the progression of running performance is supported by direct comparisons between the youngest (3 years) and oldest (6 years) groups in key parameters for both sexes, demonstrating a clear developmental trend. Among the spatiotemporal changes (Table 1), two parameters were most notable: running speed and step length. Running speed increased in both boys and girls, from 2.9 m/s to 3.9 m/s in boys (Δ +34.5%) and from 2.8 m/s to 3.8 m/s in girls (Δ +35.7%). Step length also increased, from 68.3 cm to 92.0 cm in boys (Δ +34.7%) and from 70.7 cm to 93.3 cm in girls (Δ +32.0%). These results are partially comparable with previous findings, showing a linear increase in walking speed (1.05 m/s to 1.25 m/s, Δ +19.0%) and step length (68.6 cm to 79.8 cm, Δ +16.3%, *p* < 0.05) in 383 Spanish preschool children aged 3–5 years (207 girls, 176 boys) [24], and with reports of significant age-related increases in walking speed and step length in 64 children aged 2, 3, and 6 years (*p* < 0.05) [22]. Although these studies measured walking rather than running, they confirm a general developmental trend toward increased biomechanical efficiency. Running performance in preschool children, including 15 children aged 3–4 years and 15 children aged 5–6 years, showed that older preschoolers ran significantly faster than younger children [14]. Similarly, age-related decreases in 20-m sprint times were observed in a sample of 3076 children, indicating faster running with increasing age [12]. Using their data, we calculated running speed by dividing the distance (20 m) by the average time achieved by children of different ages, revealing that average speed increased with age: 2.89 m/s at 3 years, 3.21 m/s at 4 years, 3.45 m/s at 5 years, and 3.64 m/s at 6 years, representing an overall improvement of 26%. Children aged 3–6 years therefore demonstrate substantial developmental progress in spatiotemporal running characteristics, primarily reflected in increased speed and step length in both sexes.

The development of running between ages three and six is shaped by the concurrent maturation of neurological processes and the biomechanical organization of movement. From a physiological perspective, early childhood is characterized by rapid increases in white matter microstructural integrity, particularly through progressive myelination, which enhances signal conduction velocity within motor pathways and results in more efficient neuromuscular activation [24]. This neurological maturation supports advances in intersegmental coordination, reductions in movement variability, and improved management of inertial forces during locomotion—features that Dewolf et al. [25]. describe as a transition from early, highly variable, and energetically less efficient movement patterns towards increasingly coordinated and functionally economical gait. Empirical evidence shows that these developmental changes are already clearly observable within the preschool years. Plesek et al. [14] found that children aged 5–6 years display a more mature loading profile during running, with lower vertical impact forces and greater stability during foot–ground interaction compared with children aged 3–4 years. These findings indicate that improvements in running efficiency among older preschool children are driven by the interplay between advancing neuromuscular maturation and enhanced biomechanical regulation of movement, forming essential foundations for the emergence of an adult-like running gait.

Among the detected biomechanical changes, the proportion of the propulsive phase stood out, increasing from 43.3% to 52.3% in boys (Δ +20.8%) and from 38.2% to 50.3% in girls (Δ +31.7%). The propulsive phase, from “foot flat” to “toe-off,” is the period during which the lower limb utilizes the stretch-shortening cycle to generate horizontal and vertical forces, enhancing neuromuscular efficiency [26]. Our findings align with previous research, which reported that vertical loading rate (VILR) during push-off decreased by approximately 31% with age (*p* = 0.012, d = 1.02), reflecting improved shock absorption and push-off strength [14]. The lower VILR in older preschoolers complements our observations of longer propulsive phase durations and confirms the development of more efficient push-off biomechanics between ages 3 and 6. With growth, children gradually acquire the ability to exploit the elastic-motor response for stronger and more economical push-offs.

We then examined sex differences across three levels of running dynamics: (1) temporal-kinematic phase of the step, (2) spatiotemporal movement characteristics, and (3) propulsive phase as an indicator of muscular activity. Statistically significant differences between boys and girls were observed across all three levels, but they were not consistent across all age groups, suggesting that sex differences in early childhood are neither linear nor permanent, but rather developmentally and age-specific (Table 2).

**Table 2:** Main effects for running performance.

	Main Effects: *p* (Eta Squared)		
Study Variables	Sex	Age	Sex × Age Interaction
Contact time (s)	0.057 (0.009)	0.409 (0.007)	0.199 (0.010)
Contact time (%)	0.187 (0.005)	0.011 (0.029)	0.120 (0.016)
Flight time (s)	0.458 (0.001)	0.656 (0.004)	0.563 (0.005)
Flight time (%)	0.087 (0.008)	<0.001 (0.054)	0.094 (0.017)
Swing phase (s)	0.025 (0.026)	0.801 (0.005)	0.518 (0.012)
Stride time (s)	0.006 (0.038)	0.927 (0.002)	0.797 (0.005)
Elevation (cm)	0.084 (0.008)	0.068 (0.019)	0.432 (0.007)
Speed (m/s)	0.002 (0.025)	<0.001 (0.430)	0.974 (0.001)
Acceleration (m/s^2^)	0.115 (0.014)	0.033 (0.048)	0.274 (0.021)
Step (cm)	0.400 (0.002)	<0.001 (0.402)	0.993 (0.000)
Stride (cm)	0.643 (0.001)	<0.001 (0.164)	0.973 (0.001)
Pace (steps/s)	<0.001 (0.050)	0.373 0.008)	0.957 (0.001)
Pace (steps/min)	<0.001 (0.050)	0.377 (0.008)	0.955 (0.001)
Contact phase (s)	<0.001 (0.030)	0.842 (0.002)	0.113 (0.015)
Contact phase (%)	0.002 (0.026)	0.782 (0.003)	0.117 (0.015)
Foot flat phase (s)	0.004 (0.021)	<0.001 (0.110)	0.649 (0.004)
Foot flat phase (%)	0.091 (0.008)	<0.001 (0.099)	0.484 (0.006)
Propulsive phase (s)	0.140 (0.006)	<0.001 (0.078)	0.484 (0.006)
Propulsive phase (%)	0.010 (0.017)	<0.001 (0.105)	0.640 (0.004)

In the temporal-kinematic phase of the step (1), girls exhibited a longer swing phase (0.31 s vs. 0.29 s; *p* < 0.001), longer stride time (0.49 s vs. 0.46 s; *p* < 0.001), and a greater proportion of the contact phase in the total cycle (9.9% vs. 8.3%; *p* < 0.001), indicating a greater need for stabilization and precise movement control. The standard deviation of stride time nearly halved with age in girls (from 0.06 s to 0.03 s) but increased in boys (from 0.04 s to 0.07 s), suggesting earlier pattern homogeneity in girls. Conversely, variability in the contact phase proportion increased in both sexes (e.g., at 6 years: 10.8% girls vs. 8.3% boys), possibly reflecting experimentation with different stabilization strategies.

Our findings partially align with studies involving 161 children aged 3.5–12 years, which showed that support phase duration decreased and swing phase duration increased with age, with girls demonstrating higher pelvic symmetry, indicating earlier development of stabilization mechanisms [13]. Additionally, girls were found to consistently maintain a neutral foot support, confirming their earlier biomechanical stability [15]. From these results, we conclude that preschool girls exhibit more consistent temporal running structures and greater biomechanical stabilization than boys, likely reflecting earlier neuromotor control development.

In the analysis of spatiotemporal characteristics (2), boys achieved higher average speed (3.4 m/s vs. 3.2 m/s; *p* = 0.005) and higher step frequency (4.3 steps/s vs. 4.0 steps/s; *p* < 0.001), while step length differences were not significant, suggesting that higher speed primarily results from increased step frequency. These results indicate greater explosiveness and more intense lower-limb muscle activation in boys. This is consistent with findings showing that preschool boys outperform girls in gross motor tasks involving coordination, speed, and muscular endurance, whereas girls excel in fine motor tasks [27]. From this, we conclude that boys exhibit greater lower-limb stretch-shortening capacity, reflected in higher speed and step frequency.

The third level (3), assessing the propulsive phase as an indicator of muscular activity, showed that boys exhibited a higher proportion of the propulsive phase compared to girls (46.5% vs. 43.5%; *p* = 0.009), reflecting stronger push-off and greater explosive power. These findings are consistent with previous evidence reporting a 31% lower VILR in older children compared to younger ones, indicating more efficient force absorption and improved biomechanical control during push-off [14].

Although several statistically significant sex differences were observed, they were not consistent across all age groups. The dynamic nature of sex differences is supported by evidence emphasizing the developmental dependency of motor abilities and the importance of considering age specificity when interpreting these differences [28,29].

Analysis of interactions between sex and age did not reveal statistically significant differences (*p* > 0.05), indicating that the development of running parameters with increasing age did not differ between boys and girls. Therefore, observed differences are likely transient developmental features rather than stable characteristics.

## 5. Conclusions

The results of this study confirm that sex-related differences in running parameters are present in early childhood; however, these differences are inconsistent, age-dependent, and likely related to motor system maturity. The developmental progression of running performance between ages 3 and 6 is evident in increased speed, step length, and propulsive phase, reflecting neuromuscular and biomechanical maturation. Sex-specific patterns are observable, with boys showing higher speed and step frequency, and girls demonstrating greater temporal consistency and stabilization, yet these differences are not uniform across all ages. Substantial inter-individual variability indicates that single measurements may not reflect permanent motor patterns but rather the current developmental status of each child. Therefore, caution is warranted when interpreting sex differences in early running. A key limitation of this study is the single-time assessment of the children. Additionally, only 2-m OptoJump bars were used due to equipment limitations, which may have influenced the measurement of certain running parameters. The normative values and age-specific developmental patterns presented in this study may serve as a practical reference for early motor assessment in preschool settings. These data can help educators and practitioners identify children who deviate from typical running development, informing targeted physical education strategies and early preventive interventions. Moreover, they may contribute to the refinement of motor screening protocols and the design of evidence-based programs to promote healthy movement behaviors in early childhood. Future research should consider longitudinal monitoring of the same children, which would allow for a better understanding of the developmental dynamics of running patterns. It would also be valuable to include additional influencing factors, such as environmental conditions, body composition, frequency of physical activity, and similar variables.

## Figures and Tables

**Table 1:** Sex- and age-specific for running performance, data presented as mean (SD) or median (IQR).

Sex	Boys				Girls			
Age	3 yrs (N = 43)	4 yrs (N = 62)	5 yrs (N = 42)	6 yrs (N = 39)	3 yrs (N = 62)	4 yrs (N = 61)	5 yrs (N = 55)	6 yrs (N = 41)
Contact time (s)	0.18 (0.17–0.19)	0.17 (0.16–0.18)	0.18 (0.16–0.19)	0.17 (0.15–0.18)	0.18 (0.17–0.19)	0.18 (0.17–0.19)	0.18 (0.17–0.19)	0.18 (0.17–0.19)
Contact time (%)	77.7 (6.7)	72.8 (11.9)	73.2 (7.9)	70.3 (6.7)	73.4 (12.5)	72.0 (7.0)	71.0 (6.6)	72.7 (6.8)
Flight time (s)	0.05 (0.04–0.07)	0.06 (0.05–0.07)	0.06 (0.05–0.08)	0.07 (0.06–0.09)	0.06 (0.05–0.08)	0.07 (0.06–0.09)	0.07 (0.06–0.09)	0.07 (0.05–0.08)
Flight time (%)	22.3 (6.7)	25.6 (8.5)	26.8 (7.9)	30.0 (6.7)	25.3 (7.9)	28.0 (7.0)	29.0 (6.6)	27.3 (6.8)
Swing phase (s)	0.29 (0.03)	0.28 (0.03)	0.29 (0.04)	0.29 (0.05)	0.30 (0.04)	0.31 (0.04)	0.31 (0.03)	0.29 (0.03)
Stride time (s)	0.47 (0.04)	0.46 (0.04)	0.47 (0.06)	0.47 (0.07)	0.49 (0.06)	0.49 (0.04)	0.49 (0.04)	0.48 (0.03)
Elevation (cm)	0.4 (0.2–0.5)	0.5 (0.3–0.7)	0.5 (0.3–0.8)	0.6 (0.4–1.0)	0.5 (0.3–0.8)	0.6 (0.4–1.0)	0.6 (0.4–1.0)	0.6 (0.3–0.8)
Speed (m/s)	2.9 (0.3)	3.3 (0.4)	3.5 (0.4)	3.9 (0.4)	2.8 (0.4)	3.2 (0.3)	3.4 (0.5)	3.8 (0.4)
Acceleration (m/s^2^)	0.4 (0.1–0.8)	0.2 (−0.3–0.7)	0.9 (0.07–1.5)	0.8 (0.4–2.2)	0.1 (−0.3–0.5)	0.3 (−0.04–0.6)	0.5 (0.2–0.8)	−0.3 (−1.0–2.6)
Step (cm)	68.3 (9.4)	77.5 (8.7)	86.3 (12.2)	92.0 (13.4)	70.7 (16.2)	79.0 (9.4)	87.0 (11.4)	93.3 (9.6)
Stride (cm)	122.8 (16.9)	134.1 (16.2)	143.0 (23.3)	147.6 (25.9)	122.3 (23.7)	135.5 (18.2)	145.0 (22.1)	146.7 (22.8)
Pace (steps/s)	4.3 (0.4)	4.3 (0.5)	4.2 (0.6)	4.3 (0.7)	4.3 (0.4)	4.3 (0.5)	4.2 (0.6)	4.3 (0.7)
Pace (steps/min)	255.7 (24.2)	258.5 (29.1)	250.0 (36.7)	256.5 (43.2)	239.5 (40.5)	242.2 (25.3)	237.4 (26.4)	242.3 (29.0)
Contact phase (s)	0.01 (0.008–0.02)	0.01 (0.008–0.02)	0.02 (0.009–0.02)	0.02 (0.008–0.02)	0.02 (0.01–0.02)	0.02 (0.01–0.02)	0.02 (0.01–0.03)	0.02 (0.01–0.03)
Contact phase (%)	8.1 (3.8)	7.7 (3.9)	9.5 (5.5)	8.3 (4.8)	9.7 (4.9)	10.1 (5.1)	9.0 (4.3)	10.8 (6.0)
Foot flat phase (s)	0.09 (0.07–0.1)	0.08 (0.06–0.10)	0.08 (0.06–0.09)	0.07 (0.05–0.08)	0.08 (0.07–0.10)	0.08 (0.07–0.10)	0.08 (0.07–1.0)	0.08 (0.06–0.09)
Foot flat phase (%)	48.5 (8.9)	47.5 (13.5)	43.3 (10.1)	39.0 (12.5)	52.0 (8.7)	46.7 (9.2)	46.0 (10.5)	41.0 (11.6)
Propulsive phase (s)	0.08 (0.07–0.09)	0.08 (0.07–0.09)	0.08 (0.07–0.09)	0.09 (0.07–0.1)	0.08 (0.07–0.08)	0.08 (0.07–0.09)	0.08 (0.07–1.0)	0.08 (0.07–1.0)
Propulsive phase (%)	43.3 (8.7)	44.8 (12.6)	47.1 (10.4)	52.3 (11.9)	38.2 (6.5)	43.2 (7.6)	44.5 (9.6)	50.3 (15.9)

*p* < 0.05.

## Data Availability

The original contributions of the data analyzed in this study are included in this article. Inquiries regarding these data or the raw data can be directed to the corresponding author.

## References

[1] Tanaka C., Hikihara Y., Ohkawara K., Tanaka S. (2012). Locomotive and non-locomotive activity as determined by triaxial accelerometry and physical fitness in Japanese preschool children. Pediatr. Exerc. Sci..

[2] Petrić V., Ljubičić S., Jakšić S. (2024). Inter-individual Differences in Children’s Kinematic Patterns of Running and Their Relationship with Body Mass Index. Coll. Antropol..

[3] Nguyen T., Obeid J., Timmons B.W. (2011). Reliability of fitness measures in 3- to 5-year-old children. Pediatr. Exerc. Sci..

[4] Petrić V. (2022). Kineziološke Aktivnosti Djece Rane i Predškolske Dobi—Postignuća Kineziološke Metodike.

[5] Hardy L.L., King L., Farrell L., Macniven R., Howlett S. (2010). Fundamental movement skills among Australian preschool children. J. Sci. Med. Sport..

[6] Stodden D.F., Goodway J.D., Langendorfer S.J., Roberton M.A., Rudisill M.E., Garcia C., Garcia L.E. (2008). A developmental perspective on the role of motor skill competence in physical activity: An emergent relationship. Quest.

[7] Ahn S., Fedewa A.L. (2011). A meta-analysis of the relationship between children’s physical activity and mental health. J. Pediatr. Psychol..

[8] Janssen I., Leblanc A.G. (2010). Systematic review of the health benefits of physical activity and fitness in school-aged children and youth. Int. J. Behav. Nutr. Phys. Act..

[9] Lubans D.R., Morgan P.J., Cliff D.P., Barnett L.M., Okely A.D. (2010). Fundamental movement skills in children and adolescents: Review of associated health benefits. Sports Med..

[10] Wood A.P., Imai S., McMillan A.G., Swift D., DuBose K.D. (2020). Physical activity types and motor skills in 3-5-year old children: National Youth Fitness Survey. J. Sci. Med. Sport..

[11] Babić V. (2010). Atletika Hodanja i Trčanja.

[12] Latorre-Román P.Á., Mora-López D., Martínez-Redondo M., García-Pinillos F. (2017). Reference values for running sprint field tests in preschool children: A population-based study. Gait Posture.

[13] Gieysztor E., Kowal M., Paprocka-Borowicz M. (2021). Gait Parameters in Healthy Preschool and School Children Assessed Using Wireless Inertial Sensor. Sensors.

[14] Plesek J., Hamill J., Freedman Silvernail J., Skypala J., Jandacka D. (2022). Age differences in running biomechanics during footstrike between preschool children and adults. J. Sports Sci..

[15] Latorre-Román P.Á., Párraga-Montilla J.A., Guardia-Monteagudo I., García-Pinillos F. (2018). Foot strike pattern in preschool children during running: Sex and shod–unshod differences. Eur. J. Sport. Sci..

[16] Raffalt P.C., Alkjaer T., Simonsen E.B. (2017). Intra-subject variability in muscle activity and co-contraction during jumps and landings in children and adults. Scand. J. Med. Sci. Sports.

[17] Payne G., Peixin G., Guoli L. (2008). Introduction to Human Motor Development.

[18] World Medical Association (2013). World Medical Association Declaration of Helsinki: Ethical principles for medical research involv-ing human subjects. JAMA.

[19] Healy R., Kenny I., Harrison A.J. Estimating step parameters using photoelectric cells. Proceedings of the XXXIII International Conference of Biomechanics in Sports.

[20] Krosschell K.J., Townsend E.L., Kiefer M., Simeone S.D., Zumpf K., Welty L., Swoboda K.J. (2022). Natural history of 10-meter walk/run test performance in spinal muscular atrophy: A longitudinal analysis. Neuromuscul. Disord..

[21] Lockie R.G., Murphy A.J., Schultz A.B., Jeffriess M.D., Callaghan S.J. (2013). Influence of sprint acceleration stance kinetics on velocity and step kinematics in field sport athletes. J. Strength. Cond. Res..

[22] Rygelová M., Uchytil J., Torres I.E., Janura M. (2023). Comparison of spatiotemporal gait parameters and their variability in typically developing children aged 2, 3, and 6 years. PLoS ONE.

[23] Boobani B., Kalnina Z., Glaskova-Kuzmina T., Erina L., Liepina I. (2025). Utilizing Facial Emotion Analysis with FaceReader to Evaluate the Effects of Outdoor Activities on Preschoolers’ Basic Emotions: A Pilot Study in e-Health. Int. J. Online Biomed. Eng..

[24] Stephens R.L., Langworthy B.W., Short S.J., Girault J.B., Styner M.A., Gilmore J.H. (2020). White matter development from birth to 6 years of age: A longitudinal study. Cereb. Cortex.

[25] Dewolf A.H., Sylos-Labini F., Cappellini G., Lacquaniti F., Ivanenko Y. (2020). Emergence of different gaits in infancy: Relationship between developing neural circuitries and changing biomechanics. Front Bioeng Biotechnol..

[26] Latorre Román P.Á., Párraga Montilla J.A., Robles Fuentes A., Roche Seruendo L.E., Lucena Zurita M., Muñoz Jiménez M., Consuegra González P.J. (2022). Reference values of spatial and temporal gait parameters in a contemporary sample of Spanish preschool children: A cross-sectional study. Children.

[27] Sudlow A., Galantine P., Vercruyssen F., Peyrot N., Raymond J.J., Duché P. (2023). Which factors influence running gait in children and adolescents? A narrative review. Int. J. Environ. Res. Public. Health.

[28] Zheng Y., Ye W., Korivi M., Liu Y., Hong F. (2022). Gender differences in fundamental motor skills proficiency in children aged 3–6 years: A systematic review and meta-analysis. Int. J. Environ. Res. Public. Health.

[29] Kokštejn J., Musálek M., Tufano J.J. (2017). Are sex differences in fundamental motor skills uniform throughout the entire preschool period?. PLoS ONE.

